# Teaching Neuroscience: A Primer for Psychotherapists

**DOI:** 10.3389/fnbeh.2018.00307

**Published:** 2018-12-11

**Authors:** Deborah L. Cabaniss

**Affiliations:** Department of Psychiatry, Columbia University, New York City, NY, United States

**Keywords:** psychotherapy education, psychotherapy training, psychoanalysis, unconscious, epigenetics

## Abstract

From the beginning of their psychotherapy training, students need to think about how talking changes the brain, how development is encoded in the body, and how connecting neuroscience and psychotherapy can help us improve psychosocial interventions to optimally help patients. But teaching neuroscience doesn't come naturally to many psychotherapy educators—myself included. We were trained as clinicians, not as researchers, so for many of us, reading and searching the neuroscience literature is challenging. Over many years, and with the help of wonderful colleagues, I am learning to read neuroscience papers and to incorporate what I learn into my psychotherapy teaching.

When I teach neuroscience in a psychotherapy course, I do it with great humility. I make it very clear to my students that I'm not a neuroscientist and that I'm not an expert in the field. Instead, I learn *with* my students, as together we try to understand the science and what it can tell us about the mind, development, and psychotherapy.

I also make it very clear that I'm not presenting this material as if it proves something about psychotherapy. We don't know enough about the neuroscience of psychotherapy to do that. Rather, I'm trying to get my students as excited as I am about what neuroscience can teach us about psychotherapy. My hope is that it will stimulate them to think about connections between neuroscience and psychotherapy when they are talking to patients, thinking about formulation, conceptualizing experiments and choosing their careers.

Over the years, I've found that using carefully chosen neuroscience papers that *I* can understand really helps me to get the neuroscience/psychotherapy conversation going in a classroom. To that end, I offer five papers that I use when I teach psychotherapy. They are all written by top researchers and published in the nation's premiere scientific journals. Each one provides interesting potential insights into a different aspect of psychotherapy.

## Psychotherapy Changes the Brain

Eric Kandel—Psychotherapy and the Single Synapse

The first paper I give my second-year residents to read in their Introduction to Psychodynamic Psychotherapy Course isn't by Freud, Kohut, or even Kernberg. It's by Eric Kandel and it's called “Psychotherapy and the Single Synapse” (Kandel, 1979). It was published in the *New England Journal of Medicine* in 1979. When I first read this paper as a resident, it blew my mind. Here was Eric Kandel, who had taught me neuroscience in medical school, written the neuroscience textbook I had read, and was soon to win the Nobel Prize in Physiology or Medicine, writing about psychotherapy. Who knew that he had any interest in that? As I later learned from reading his award-winning memoir, “In Search of Memory” (Kandel, 2006), Dr. Kandel was born in Vienna and has had a longstanding interest in psychoanalysis. The very fact that the most famous neuroscientist in my department was writing about psychotherapy was significant to me. And it wasn't even published in a psychiatry journal—it was published in the *New England Journal of Medicine*! Even at that first reading, I could feel the concepts of mind and brain coming together, and the artificial dichotomy between functional and organic dissolving. And today, in 2018, it still has that effect on my residents.

In this brilliant, prescient, paper, Kandel talks about himself as a young psychiatry resident at the Massachusetts Mental Health Center in 1960 (it's an added extra that he identifies as a clinician), grappling with his colleagues about whether neuroscience was important for understanding psychiatric illness. In his characteristic clear, persuasive style, he takes the reader through his argument that, in fact, it is. Reviewing studies by Rene Spitz, Harry Harlowe, and Hubel and Wiesel, as well as his own work on the physiologic underpinnings of learning, he argues that since both early sensory deprivation and later learning have been shown to have longstanding, lasting effects on the brain, the same must be true of psychotherapy. “Ultimately, all psychologic disturbances reflect specific alterations in neuronal and synaptic function,” he writes. “And insofar as psychotherapy works, it works by acting on brain functions, not on single synapses, but on synapses nevertheless.” He goes on to say:

…*when I speak to someone and he or she listens to me, we not only make eye contact and voice contact but the action of the neuronal machinery in my brain is having a direct and, I hope, long-lasting effect on the neuronal machinery in his or her brain…Indeed, I would argue it is only insofar as our words produce changes in each other's brains that psychotherapeutic intervention produces changes in patients' minds*.

Bottom line: psychotherapy is a brain-changer. That's the message I want to convey to my students as they begin to learn psychotherapy, and there's nothing like this classic paper to help me do that.

## The Unconscious is in the Brain

Antoine Bechara et al—Deciding Advantageously Before Knowing the Advantageous Strategy

In 1895, Sigmund Freud, wrote his “Project for a Scientific Psychology” at white heat, eager to explain his new psychological findings as having their basis in the substrate of the nervous system (Freud, 1950). But he abandoned it unfinished, moving forward with a psychology unrooted in the brain. Why? Was it because he thought he was wrong? Hard to imagine that this young physician, trained in physiology and neurology, was truly leaving behind the newly described neuron. As Kandel explains in “Single Synapse,” until recently, neurobiology was not mature enough to shed light on “higher order” psychological functions (Kandel, 1979). But that's not necessarily true anymore.

Since psychodynamic psychotherapy is based on the idea that unconscious elements and processes affect conscious function, there's no better entry point to discussing the way that psychoanalytic functions could be produced by the brain than the concept of the unconscious. And there's no better paper to facilitate that conversation than “Deciding Advantageously Before Knowing the Advantageous Strategy,” written by Antoine Bechara, Hanna Damasio, Daniel Tranel and Antonio Damasio and published in *Science* in 1997 (Bechara et al., 1997).

Again, this isn't just any paper, it's a paper from Damasio's lab published in *Science*. In it, the investigators describe an experiment in which they ask two groups of subjects—one with normal brain function and one with prefontal damage and decision-making deficits—to play a gambling game in which they choose randomly from 4 decks of cards with the goal of making as much (play) money as possible. Decks A and B have cards marked with high rewards and high penalties, and Decks C and D have cards marked with lower rewards but similarly lower penalties. Winning requires choosing from Decks C and D. The subjects were given no information about the decks and were instructed to choose cards randomly. As the game went on, subjects were periodically asked what they understood about the game and were also monitored for anticipatory skin-conductance responses. Normal subjects began choosing advantageously before they understood why, suggesting that their choices were guided by what the authors call non-conscious biases (aka unconscious processes). In addition, only normals developed these “hunches,” suggesting that these non-conscious biases are generated in the prefrontal cortex.

This is a terrific paper to use in a psychotherapy course for many reasons. It has neuroscientists investigating properties of the unconscious and suggests some type of localization for at least this unconscious function. It's also a classic cognitive neuroscience paper, insofar as it uses patients with a localized deficit to demonstrate something about the function of a brain area. You don't need to be able to read scans to understand it. It's also great to teach using the cognitive neuroscience literature, since, at this point, studies conducted by cognitive neuroscientists connect to psychotherapy more readily than most circuit, synaptic, cellular, or molecular level studies. The need to translate from “non-conscious bias” to “unconscious” is also helpful, in that it will help students decode this in other papers. Plus, it's two pages long, well-written, and about gambling—perfect for your psychotherapy syllabus.

## Psychotherapy and Memory

Daniella Schiller et al—Preventing the return of fear in humans using reconsolidation update mechanisms

“*Hysterics suffer mainly from reminiscences*,” wrote Freud and Breuer in 1893 (Breuer and Freud, 1893). That finding led the two men to their discovery of psychotherapy—the talking cure—designed to help people alleviate symptoms by talking about repressed memories. Although we now know that it's more complicated than that, memory and talking about memories is at the heart of psychodynamic psychotherapy—and it seems clear that something about talking about memories in therapy is therapeutic. But why? Although we don't yet know, scientists are actively working to understand how memory works and how retrieving memories can be therapeutic.

I often introduce this topic with the paper, “Preventing the return of fear in humans using reconsolidation update mechanisms,” featuring experiments from the NYU lab of Elizabeth Phelps and published in *Nature* in 2010 (Schiller et al., 2010). In this paper, lead author Daniella Schiller describes an experiment on humans, based on the concept of memory reconsolidation. Although memory was originally thought to be a one-time process, scientists working with animal models have shown that memories change every time they are remembered, and that this process requires protein synthesis (Alberini, 2011). The idea is that, during the reconsolidation, the memory is “labile” and thus potentially vulnerable to change. To test this, Schiller and her colleagues created a fear memory in three groups of people—a mild shock connected to a color—then brought them back a day later to try to extinguish the memory. For two of the groups, they preceded the extinction with a reminder of the fear memory (the color), presenting this reminder 10 min prior to extinction in one group and 6 h prior to extinction in the other. The group that received the reminder 10 min before did the best—and the extinction lasted up to a year.

To me, this paper offers a great entrée into a discussion of how talking about memories might alter them. This happens in all types of psychotherapy, from CBT to psychoanalysis. This paper helps to foster discussion of what actually happens when we talk about memories in psychotherapy. The idea that we stir up a memory and then work with it during a period of lability could shed light on the way that psychotherapy helps people think differently about a parent, or revise their sense of self. Students often ask me why we recommend that patients in psychodynamic psychotherapy come more than once a week—this paper actually makes me think that it might be better to see someone in the morning and then again after lunch!

## How is Early Environmental Experience Encoded in the Body?

Michael Meaney—Maternal Care, Gene Expression and the Transmission of Individual Differences in Stress Reactivity Across Generations

Sabine Herpertz and Katja Bertch—A New Perspective on the Pathophysiology of Borderline Personality Disorder: A Model of the Role of Oxytocin

As a psychoanalyst, there's nothing more exciting than studies investigating how early environmental experiences are encoded in the body. Learning about these alongside psychodynamic developmental theories enriches students' ideas about formulation and deepens their understanding of their patients. A great place to start this conversation is with epigenetics –the study of gene modification that occurs due to factors other than direct modification of the genetic code. In his paper, “Maternal Care, Gene Expression and the Transmission of Individual Differences in Stress Reactivity Across Generations,” published in the *Annual Review of Neuroscience* (Meaney, 2001), Michael Meaney reviews the findings of his lab and others that, rat pups who receive more nurturing from their mothers (which translates into more licking and grooming) have decreased stress reactivity than pups who receive less. The really exciting finding is that this seems to be mediated by differential methylation of histones—the proteins around which DNA is wound in the cell nucleus. Thus, early parenting directly translates into histone methylation, which mediates gene expression—and when the gene is for the glucocorticoid receptor, the connection between good parenting and later life stress response becomes strikingly clear.

A second paper on this topic is Sabine Herpertz and Katja Bertch's 2015 “A New Perspective on the Pathophysiology of Borderine Personality Disorder: A Model of the Role of Oxytocin,” published in the *American Journal of Psychiatry* (Herpertz and Bertch, 2015). Like Meaney, Herpertz and Bertch are hypothesizing about how early experience affects later behavior—here, specifically, characteristics of borderline personality disorder (BPD). They discuss the cycle in which oxytocin levels predict parental physical affection (touching and cuddling), parental care predicts childhood oxytocin levels, and childhood oxytocin levels predicts capacity for social interactions. They then review the evidence that oxytocin may mediate the triad of affect dysregulation, behavior dyscontrol, and interpersonal hypersensitivity, suggesting that oxytocin levels could be the biological mediator that translates early trauma and neglect into characteristics of BPD.

Both of these reviews are clear and seem to have been written with the clinician in mind. They are well-suited to classes on formulation and discussions of “how are patients came to be the way they are.”

## Bringing these Papers to Life in Psychotherapy Class

The findings covered in these papers are exciting and directly relevant to discussions about development, formulation, and psychotherapy. They don't have answers; rather, they spark questions. That's the spirit in which I use them with students. I choose them carefully—no more than one per class—and assign them alongside psychotherapy readings. For example, we might read the article about oxytocin alongside one by Kernberg when studying BPD. In a 1 h seminar, I don't spend a lot of time reviewing the article—either I do a brief review or I ask a student to do this—and then we ask the central question:

How do the findings in this article affect the way that you think about your patients and your work with them?

This is really what I want my students to consider. It's difficult to change the behavior of a borderline patient—could that be because we're having to reverse the methylation of histones? Should we decrease the time between sessions in order to facilitate memory recall? How should our psychotherapeutic work change when working with patients with prefrontal deficits? How could psychotherapists work with neuroscientists to learn more about the mind and how to optimize psychotherapeutic interventions?

Including neuroscience in a psychotherapy curriculum helps to break down silos by modeling that psychotherapists are interested neuroscience, actively teaching these disciplines side by side, and fostering future collaboration. So, psychotherapy educators—be brave! Stick your toe in the neuroscience literature and bring your students with you. It's easier than you might imagine, fascinating, and might even contribute to the future of psychotherapy.

## Author Contributions

The author confirms being the sole contributor of this work and approved it for publication.

### Conflict of Interest Statement

The author declares that the research was conducted in the absence of any commercial or financial relationships that could be construed as a potential conflict of interest.
